# R-carvedilol, a potential new therapy for Alzheimer’s disease

**DOI:** 10.3389/fphar.2022.1062495

**Published:** 2022-11-24

**Authors:** Jinjing Yao, S. R. Wayne Chen

**Affiliations:** ^1^ Department of Physiology and Pharmacology, Cumming School of Medicine, Libin Cardiovascular Institute, University of Calgary, Calgary, AB, Canada; ^2^ Department of Physiology and Pharmacology, Cumming School of Medicine, Hotchkiss Brain Institute, University of Calgary, Calgary, AB, Canada

**Keywords:** carvedilol, R-carvedilol, Alzheimer’s disease, RyR2, neuronal hyperactivity

## Abstract

For decades, the amyloid cascade hypothesis has been the leading hypothesis in studying Alzheimer’s disease (AD) pathology and drug development. However, a growing body of evidence indicates that simply removing amyloid plaques may not significantly affect AD progression. Alternatively, it has been proposed that AD progression is driven by increased neuronal excitability. Consistent with this alternative hypothesis, recent studies showed that pharmacologically limiting ryanodine receptor 2 (RyR2) open time with the R-carvedilol enantiomer prevented and reversed neuronal hyperactivity, memory impairment, and neuron loss in AD mouse models without affecting the accumulation of *ß*-amyloid (Aβ). These data indicate that R-carvedilol could be a potential new therapy for AD.

## Introduction

As the most common form of dementia, Alzheimer’s disease (AD) is afflicting an increasing number of people around the world. (Rocca et al., 1990; Hynd et al., 2004). In addition to plaguing its patients, AD has also become a heavy burden on patients’ families and society (Prigerson, 2003; Castro et al., 2010). Over the past three decades, scientists across the world have made immense efforts to comprehend the pathogenesis of AD and develop effective AD treatments (Corbett et al., 2012; Elmaleh et al., 2019; Clement et al., 2020). The leading theory of AD pathogenesis is the amyloid cascade hypothesis. Based on this hypothesis, most efforts have been devoted to the development of drugs targeting *ß*-amyloid (Aβ) (Demattos et al., 2012; Kennedy et al., 2016; Sevigny et al., 2016). Unfortunately, most Aβ-targeted AD clinical trials have failed to yield convincing results (Mehta et al., 2017; Yiannopoulou et al., 2019; Knopman et al., 2021). These disappointing outcomes indicate that our understanding of AD pathogenesis is far from complete, and we urgently need new strategies for AD treatment.

Given the multifactorial nature of AD pathogenesis, multiple pathways besides Aβ metabolism could be targeted for AD treatment. Indeed, recent studies have revealed many new targets and strategies for treating AD. For example, a molecule named leuco-methylthioninium bis(hydromethanesulphonate) (LMTM) that is thought to block the aggregation of tau has been shown to possess some promising anti-AD properties in a phase III clinical trial (Wilcock et al., 2018). Furthermore, recent studies suggested the ε allele of apolipoprotein E (APOE) is another genetic risk factor of AD. By injecting the anti-human APOE antibody into the APOEε4^+/+^ mouse model, Xiong et al. (2021), showed that this APOE immunotherapy reduced cerebral Aβ plaques and amyloid angiopathy. Besides focusing on certain molecular targets, more and more studies showed that neuronal circuitries could also be a promising target for treating AD. The gamma oscillations, which are important for the storage and maintenance of memory, were impaired in both AD patients and mouse models (Adaikkan and Tsai, 2020; Andrade-Talavera et al., 2020). Andrade-Talavera and colleagues found that small molecule compounds or the molecular chaperone Bri2 BRICHOS reversed the impaired gamma oscillations related to AD (Andrade-Talavera et al., 2020; Andrade-Talavera et al., 2022). In addition, multiple studies form Dr. Li-Hui Tsai’s group have shown that the gamma entrainment using sensory stimuli (GENUS) could reduce the Aβ level, improve learning and memory in multiple AD mouse models (Iaccarino et al., 2016; Adaikkan et al., 2019; Martorell et al., 2019).

In addition to these new trends in AD treatment, substantial evidence indicates that soluble Aβ induces neuronal hyperactivity, which in turn generates more soluble Aβ. These two components form a vicious cycle (Kamenetz et al., 2003; Cirrito et al., 2005; Busche et al., 2012; Yamamoto et al., 2015; Keskin et al., 2017; Zott et al., 2019). This Aβ-hyperactivity cycle is thought to promote Aβ accumulation, increase neuronal excitability, induce circuit dysfunction and exacerbate AD progression (Busche and Konnerth, 2015; Stargardt et al., 2015; Busche and Konnerth, 2016; Zott et al., 2019). Given the failure of many Aβ-targeted AD clinical trials, it seems that simply reducing Aβ expression or removing Aβ plaques is not sufficient to break this vicious cycle. Another way to break this loop is to overcome the neuronal hyperactivity.

Carvedilol is a nonselective *ß*-adrenergic receptor blocker, widely used for the treatment of congestive heart failure (Feuerstein and Ruffolo, 1995; Frishman, 1998; Dulin and Abraham, 2004). A previous study suggested that *ß*-blockers such as carvedilol may have anti-inflammatory effects on the AD brain, while it may also improve cognitive function in AD patients by improving cerebral perfusion (Rosenberg et al., 2008). However, since its potent *ß*-blocking activity that can cause bradycardia and hypotension, the benefits of carvedilol have been shown to be dose-limited (Bristow et al., 1996; Zhang et al., 2015). The clinically used carvedilol is a racemic mixture of *ß*-blocking S-carvedilol and non-β-blocking R-carvedilol (Bristow et al., 1996; Frishman, 1998; Zhou et al., 2011; Zhang et al., 2015). Recently, it has been shown that R-carvedilol alone prevented and rescued neuronal hyperactivity, memory impairment, and neuron loss without affecting the accumulation of *ß*-amyloid (Aβ) in mouse models of familial AD (FAD) *in vivo* and *in vitro* (Yao et al., 2020; Liu et al., 2021; Sun et al., 2021). This mini-review will discuss the potential application of R-Carvedilol as a new therapeutic strategy to treat AD.

### Carvedilol and R-carvedilol

Carvedilol is currently used in the form of racemic mixture, containing an equal amount of R-carvedilol (an *α*-blocker) and S-carvedilol (an *α*- and *β*-blocker). *In vitro* studies have shown that S-carvedilol has approximately 100-fold greater affinity for adrenergic *ß*-receptors than R-carvedilol. Meanwhile, R-carvedilol and S-carvedilol inhibit adrenergic *α*-receptors to the same extent (Nichols et al., 1989; Bartsch et al., 1990). Consistent with these studies, a randomized, double-blind, placebo-controlled study performed by Stoschitzky et al. (2001), indicated that only S-carvedilol causes *ß*-blockade. These findings suggest that the strong *ß*-blockade caused by S-carvedilol may underlie carvedilol’s adverse effects of bradycardia and hypotension.

Besides blocking the adrenergic receptors, carvedilol has been shown to effectively suppress spontaneous calcium release, also known as store-overload-induced calcium release (SOICR). Single channel recordings revealed that carvedilol can directly shorten the open time of the ryanodine receptor 2 (RyR2) Ca^2+^ release channel (Zhou et al., 2011). Like the racemic carvedilol, the non-β-blocking R-carvedilol can also directly reduce the open duration of RyR2 and suppress stress-induced ventricular tachyarrhythmia (VT) in mice harboring a RyR2 mutation (RyR2-R4496C^+/-^) associated with catecholaminergic polymorphic ventricular tachycardia (CPVT). Importantly, RyR2-R4496C^+/-^ mice that received R-carvedilol treatment did not show a significant change in heart rate or blood pressure (Zhou et al., 2011; Zhang et al., 2015).

### Carvedilol has protective effects on neurons

In addition to its benefits in the heart, carvedilol has also been shown to have neuroprotection property. Studies with cultured neurons suggest that carvedilol protects neuron from cell death induced by cerebral ischemia or stroke (Lysko et al., 1992; Yamagata et al., 2004). Using rat brain homogenate, Yue et al., have shown that compared to other commonly used *ß*-blockers, carvedilol is a far more potent antioxidant. The antioxidant effect of carvedilol mainly resides in the carbazole moiety, and the substitution of a hydroxyl group at certain positions on the phenyl ring of either carbazole or the ortho-substituted phenoxylethylamine part of carvedilol resulted in an increase in antioxidant activity (Yue et al., 1992). Thus, carvedilol’s actions on scavenging free radicals and inhibiting lipid peroxidation are believed to be the mechanism underlying neuroprotection. More importantly, Wang et al. (2011), reported that chronic oral administration of carvedilol in 2 independent AD mouse models, TgCRND8 transgenic mice and Tg2576 AD transgenic mice, remarkably reduced the expression of oligomeric Aβ and reversed cognitive decline (Arrieta-Cruz et al., 2010). Further studies suggested that carvedilol has the potential to bind to Aβ, thus preventing Aβ aggregation and formation of Aβ oligomeric fibrils (Wang et al., 2011). Therefore, in addition to treating congestive heart failure, carvedilol may potentially be used for treating AD or other neurological diseases. Interestingly, RyR2, a target of carvedilol, is abundantly expressed in both the heart and the brain (Furuichi et al., 1994; Giannini et al., 1995; Murayama and Ogawa, 1996; Bers, 2002). In particular, RyR2 is abundantly expressed in the hippocampus and cortex, which are the regions most vulnerable to damage caused by AD (Yao et al., 2020; Hiess et al., 2022). Increasing evidence suggests that the expression and function of RyR2 are upregulated in animal models of familial AD (FAD) and in human AD patients (Kelliher et al., 1999; Smith et al., 2005; Bruno et al., 2012; Oules et al., 2012; Chakroborty and Stutzmann, 2014; Lacampagne et al., 2017; SanMartin et al., 2017; Stutzmann, 2021). Since R-carvedilol can significantly reduce the open duration of RyR2 and does not have the *ß*-blocking effect, it represents a promising treatment for AD without the adverse effects often associated with racemic carvedilol, such as bradycardia and hypotension.

### R-carvedilol treatment reverses the increased RyR2 activity in CA1 pyramidal neurons of 5xFAD^+/-^ mice

Increasing evidence has shown that RyR2-mediated calcium release can regulate membrane excitability of various cells, such as cardiomyocytes, smooth muscles, and neurons (Nelson et al., 1995; Alkon et al., 1998; Bogdanov et al., 2001; Mandikian et al., 2014). Besides causing problems in the heart, enhanced RyR2 function is also involved in AD pathogenesis (Kelliher et al., 1999; Smith et al., 2005; Bruno et al., 2012; Oules et al., 2012; Chakroborty and Stutzmann, 2014; Lacampagne et al., 2017; SanMartin et al., 2017). Consistent with these findings, two-photon calcium imaging of mouse hippocampal slices revealed significantly greater caffeine-induced calcium release in CA1 pyramidal neurons of 5xFAD^+/-^ mouse compared with age-matched wild-type (WT) littermates. Notably, compared to DMSO-treated control group, pre-treatment with R-carvedilol for one-month, markedly reduced caffeine-induced calcium release in CA1 pyramidal neurons from acute 5xFAD^+/-^ mice brain slices (Yao et al., 2020). Therefore, R-carvedilol is able to prevent AD-induced abnormal activation of RyR2-mediated calcium release.

### R-carvedilol prevents and reverses neuronal hyperactivity in 5xFAD^+/-^ mice, *in vivo* and *ex vivo*

Previous studies with different AD mouse models have shown that hyperactivity of hippocampal CA1 pyramidal neurons is associated with AD pathogenesis (Brown et al., 2011; Kerrigan et al., 2014; Siskova et al., 2014; Scala et al., 2015). By performing *in vivo* two-photon imaging in mice expressing the Thy-1 promoter-driven GCaMP6f calcium sensor in glutamatergic neurons driven (Chen et al., 2012), Yao et al. (2020), detected increased neuronal activity in glutamatergic pyramidal neurons in the hippocampal CA1 region in anesthetized 5xFAD^+/-^ mice. Consistent with these previous observations (Busche et al., 2008; Busche et al., 2012; Busche and Konnerth, 2015, Busche and Konnerth, 2016; Zott et al., 2019), CA1 glutamatergic pyramidal neurons of 5xFAD^+/-^ mice exhibited a significantly increased proportion of hyperactive neurons and the mean frequency of spontaneous calcium transients, and also a considerably decreased proportion of normal neurons, compared with WT littermates, (Yao et al., 2020). Interestingly, recent studies with mouse brain slices treated with recombinant Aβ_1–42_ or with brain slices from the novel *App* ^NL−G-F^ mouse model showed that the gamma oscillation was significantly impaired (Balleza-Tapia et al., 2018; Andrade-Talavera et al., 2021; Arroyo-Garcia et al., 2021). Taken these studies together, it is possible that under the AD condition, the firing rates of excitatory neurons and inhibitory interneurons were altered differently. In other words, the excitatory/inhibitory balance was disrupted. Also, the change of the excitatory/inhibitory balance is dynamic, which means during the development of AD, the overall brain activity could be hyperactive at early stages and hypoactive at late stages (Targa Dias Anastacio et al., 2022).

Yao et al. (2020), also tested whether R-carvedilol treatment can prevent or reverse the hyperactivity of CA1 neurons in the 5xFAD^+/-^ mice. 5xFAD^+/-^ mice 2–3 months old (i.e., before the onset of AD symptoms) and 3–4 months old (i.e., after the onset of AD symptoms) (Oakley et al., 2006) were pre-treated with R-carvedilol or DMSO for one month. Compared to the DMSO pre-treated mice at both ages, the CA1 pyramidal neurons in 5xFAD^+/-^ mice receiving R-carvedilol treatment showed a significant decrease in the proportion of hyperactive neurons with an apparent reduction of the mean frequency of spontaneous calcium transients. These observations suggested that R-carvedilol pre-treatment not only prevented but also reversed neuronal hyperactivity of 5xFAD^+/-^ hippocampal CA1 neurons *in vivo* (Yao et al., 2020). Similar results were also found from *ex vivo* recordings with 5xFAD^+/-^ hippocampal slices (Sun et al., 2021). Thus, both *in vivo* and *ex vivo* recordings showed that R-carvedilol treatment before the onset of AD pathologies can prevent neuronal hyperactivity, while R-carvedilol treatment after the onset of AD pathologies can rescue neuronal hyperactivity in 5xFAD CA1 neurons.

### R-carvedilol prevents and rescues memory loss in Alzheimer’s disease mouse models

As a disease characterized primarily by cognitive impairment, a fundamental readout for any intervention is the measure of learning and memory. To test whether R-carvedilol treatment can protect animals from AD-related learning and memory deficits, Yao et al., performed the Morris water maze (MWM) test and novel object recognition (NOR) test, together with measurement of hippocampal CA3-CA1 long-term potentiation (LTP). Similar to the results of calcium imaging, pretreatment with R-carvedilol in 2–3 months old 5xFAD^+/-^ mice (before AD symptoms) prevented memory loss and reversed LTP deficit, while in 3–4 months old 5xFAD^+/-^ mice (after AD symptoms), pretreatment with R-carvedilol also reversed these deficits. The drug was also tested on 6–7 and 10–12-month-old 5xFAD^+/-^ mice to further assess whether R-carvedilol pretreatment remains effective in advanced AD stages. Results from behavioral tests and LTP recordings also suggested that pretreatment with R-carvedilol could still reverse memory deficits even in 6–7 months and 10–12 months old 5xFAD^+/-^ mice (i.e., late AD). (Yao et al., 2020).

As mentioned above, previous studies suggested that the racemic carvedilol may reduce the expression of oligomeric Aβ and prevent plaque formation and cognitive decline in multiple mouse models of AD (Arrieta-Cruz et al., 2010; Wang et al., 2011). However, Yao et al. (2020), showed that racemic carvedilol did not prevent cognitive decline in 3–4 months old 5xFAD^+/-^ mice (Yao et al., 2020). This new finding may help us to explain the failure of a recent AD clinical trial with the racemic mixture of carvedilol (https://www.clinicaltrials.gov/ct2/show/study/NCT01354444). The exact reason for the ineffectiveness of the carvedilol racemic mixture in inhibiting AD progression is unknown. One possibility is that the strong *ß*-blocking effect of S-carvedilol in the racemic mixtures, especially at high doses, may adversely affect neuronal activity and cognitive function, thus counteracting the beneficial effects of R-carvedilol. Furthermore, the beneficial effect of R-carvedilol on learning and memory is dose-dependent. R-carvedilol at a dose of 3.2 mg/kg/day or 1.6 mg/kg/day, but not 0.8 mg/kg/day, rescued cognitive decline in 5xFAD mice.

In the study of Yao et al. (2020), they employed the 5xFAD mouse as the animal model of AD. Based on the original study of 5xFAD mice, this model contains 5 human FAD mutations. Thus, in addition to reproducing the prominent symptoms found in AD patients, it significantly shortens the latency to onset of AD-related pathological features (Oakley et al., 2006), significantly reducing the time period of experimental studies. However, this fast onset of AD pathology in 5xFAD mice is very different from that in human AD (Lee and Han, 2013; Jankowsky and Zheng, 2017). To determine whether R-carvedilol can also prevent and rescue cognitive decline in a mouse model whose AD progression is relatively slower than the 5xFAD mouse, Liu et al. (2021), employed the well-known 3xTG AD mouse model, which is also widely used in AD studies, but the onset of AD symptoms is relatively late (Oddo et al., 2003; Jankowsky and Zheng, 2017). 3xTG^+/-^ mice (12–15 months old) were treated with R-carvedilol or DMSO for one month and behavioral tests and LTP measurements were conducted. Similar to the studies with 5xFAD mice, Liu et al. (2021) showed that in the training session of the MWM test, compared to the 3xTG^+/-^ mice pretreated with DMSO, 3xTG^+/-^ mice with R-carvedilol pre-treatment spent significantly less time to find the target platform. While in the probe trials, 3xTG^+/-^ mice that received R-carvedilol pre-treatment spent significantly more time in the area where the platform used to be. Interestingly, mice received R-carvedilol pre-treatment also showed increased speed of swimming compared to the DMSO control group. Similarly, results from the NOR test suggested R-carvedilol pre-treatment increased the discrimination index and the walking velocity in the 3xTG^+/-^ mice. LTP recordings also suggested that R-carvedilol pre-treatment significantly improved the neuronal circuit function in 3xTG^+/-^ mice (Liu et al., 2021). In light of these observations, it would also be of interest to assess whether R-carvedilol could prevent cognitive decline in other AD mouse models, such as the *App* ^NL−G-F^ mouse model, the *App* ^NL/NL^ and *App* ^NL−F/NL−F^ mouse models, and the APOEε4^+/+^ mouse model (Saito et al., 2014; Andrade-Talavera and Rodriguez-Moreno, 2021; Arroyo-Garcia et al., 2021; Xiong et al., 2021).

### R-carvedilol protects against neuronal cell death but does not affect Aβ accumulation in Alzheimer’s disease mouse models

Neuronal cell death is one of the clinicopathological features observed from human AD patients (Shimohama, 2000; Niikura et al., 2006; Goel et al., 2022), but neuron loss in AD transgenic mouse models is controversial (Wirths and Bayer, 2010; Wirths and Zampar, 2020). Consistent with previous reports (Oakley et al., 2006; Jawhar et al., 2012), Yao et al. (2020) showed that in the subiculum region of the hippocampus, the number of pyramidal neurons was significantly reduced in both aged 5xFAD^+/-^ and 3xTG^+/-^ brain slices (Liu et al., 2021). Notably, R-carvedilol pretreated 5xFAD^+/-^ and 3xTG^+/-^ mice showed a significantly higher density of neurons in the subiculum area compared to the DMSO-pretreated mice. These results suggested that, like carvedilol, R-carvedilol may also have the ability to protect neurons from neuronal cell death. Besides neuronal hyperactivity, learning and memory impairment, and neuronal cell death, accumulation of aggregated Aβ in the brain is believed to be one of the major hallmarks of AD (Bharadwaj et al., 2009; Murphy and LeVine, 2010; Chen et al., 2017). R-carvedilol treatment seems to be able to rescue major deficits in AD mouse models. Can it also reduce Aβ accumulation? Surprisingly, immunohistochemical staining and immunoblotting analyses of brain samples from 5xFAD^+/-^ mice or 3xTG^+/-^ mice indicated that R-carvedilol pre-treatment did not affect Aβ accumulation (Yao et al., 2020; Liu et al., 2021). Therefore, shortening RyR2 open time with R-carvedilol may represent a novel strategy for treating AD without targeting Aβ.

## Conclusion

Carvedilol has been used for treating cardiovascular diseases for decades. New indications for carvedilol are emerging. In addition to its beneficial effects on the heart, carvedilol has been shown to have potential for combatting cancer, neurological disorders, and other diseases (Lysko et al., 1992; Yamagata et al., 2004; Arrieta-Cruz et al., 2010; Wang et al., 2011; Huang et al., 2017; Chen et al., 2020; Liang et al., 2021; Abdullah Shamim et al., 2022). The list of new applications of carvedilol is expected to increase with better understanding of the mechanisms of action of carvedilol and its enantiomer, R-carvedilol. Recent studies have shown that pharmacologically shortening the open time of RyR2 with R-carvedilol prevented and reversed neuronal hyperactivity, memory impairment, and neuronal cell death in different AD mouse models without affecting the accumulation of Aβ (Yao et al., 2020; Liu et al., 2021; Sun et al., 2021). The exact mechanism(s) of these beneficial effects of R-carvedilol has not been determined, but it is likely complex and multifactorial. Nevertheless, increasing evidence suggests that in addition to targeting Aβ, R-carvedilol enantiomer brings us a novel hyperactivity-directed anti-AD therapeutic that warrants further preclinical studies and clinical trials.
