# Corrigendum: R-carvedilol, a potential new therapy for Alzheimer’s disease

**DOI:** 10.3389/fphar.2022.1125890

**Published:** 2023-01-12

**Authors:** Jinjing Yao, S. R. Wayne Chen

**Affiliations:** ^1^ Department of Physiology and Pharmacology, Cumming School of Medicine, Libin Cardiovascular Institute, University of Calgary, Calgary, AB, Canada; ^2^ Department of Physiology and Pharmacology, Cumming School of Medicine, Hotchkiss Brain Institute, University of Calgary, Calgary, AB, Canada

**Keywords:** carvedilol, R-carvedilol, Alzheimer’s disease, RyR2, neuronal hyperactivity

In the published article, there was an error in the **Acknowledgements** statement as Krembil Foundation was accidentally omitted. The corrected statement appears below.

